# 

**Published:** 1885-02

**Authors:** 


### THE CHICAGO MEDICAL JOURNAL AND EXAMINER

EDITORS:

S. J. JONES, M.D., L.L.D.

W. W. JAGGARD, A.M., M.D. HAROLD N. MOYER, M.D.

### CONTENTS.

PAGE.

TRANSLATIONS:
The Application of Cocaine to the Eye as an Anæsthetic. By Dr. Karl Koller, House Physician to the Allemeines Krankenhaus of Vienna. Translated for the CHICAGO MEDICAL JOURNAL AND EXAMINER by Boerne Bettman, M. D., Chicago.... 91
Cocaine and its Salts. Translated by Wm. M. Smith, M. D.... 157
Translations by A. Lagorio, M. D. New Facts about Antipirine.—Tannate of Cannabine.—Parasites in Cholera.—Permanent Antiseptic Medication.—The Comma-bacillus.—Researches on Hydrophobia.—Treatment of Cholera with Iodine.—Ovariotomy in a Child Twenty Months Old.—Whims of Hysteria.—Rendfleisch's Process to Study the Baccilli.—Diphtheritic Arthritis.—Inhalations of Oxygen.—Compressed Air.—Bran Baths.... 163-173

ORIGINAL COMMUNICATIONS:
I. Report of three Operations of Ovariotomy with Exhibition of Pathological Specimens. By D. A. K. Steele, M. D., President Chicago Medical Society, Professor Orthopedic Surgery, College Physicians and Surgeons of Chicago, Etc.... 101
II. The Bacilli of Syphilis and Hydrargyrum Tannicum Oxydulatum. By Dr. Joseph Zeisler, Chicago.... 113
III. Manganese as an Emmenagogue. By Franklin H. Martin, M. D., Chicago.... 119
IV. Diphtheria: Remarks on the Germ Theory, with Report of Cases under Alcoholic Treatment. By G. H. Chapman, M. D.... 128

EDITORIAL:
"Let Not the Cobbler Go Beyond His Last."... 139
New Remedies.... 140

PAGE.

Phosphides and Sulphides.... 142
Literary Notices.... 142
Errata.... 143

BOOK REVIEWS:
Holden's Anatomy: A Manual of Dissection of the Human Body. By Luther Holden, late President of the Royal College of Surgeons of England; Consulting Surgeon to St. Bartholomew's and the Foundling Hospital. Fifth Edition. Edited by John Langton, Surgeon to and Lecturer on Anatomy at St. Bartholomew's Hospital; Member of the Board of Examiners, Royal College of Surgeons, England. Octavo, pp. xix, 886. Philadelphia: P. Blackiston, Son & Co. 1885. Chicago: Jansen, McClurg & Co.... 144
Lectures on the Principles of Surgery. Delivered at Bellevue Hospital Medical College. By W. H. Van Buren, M. D., LL. D. (Yalen), formerly Professor of the Principles and Practice of Surgery in the Bellevue Hospital Medical College, etc., etc. Edited by Lewis A. Stimson, M. D., Professor of Physiology and Clinical Surgery in the Medical Department of the University of the City of New York. Octavo, pp. vii, 588. New York: D. Appleton & Co. 1884. Chicago: Jansen, McClurg & Co.... 144

FOREIGN CORRESPONDENCE:
Letter from Italy.... 146
Letter from Edinburgh.... 151

SELECTIONS:
Pennsylvania Anatomical Act.... 174

LOCAL NEWS:
Rush Medical College Commencement.... 176

SOCIETY PROCEEDINGS:
Chicago Gynæcological Society.... 177

